# Establishing a biomarker set for the diagnosis of apple replant disease using qPCR

**DOI:** 10.1093/hr/uhag140

**Published:** 2026-04-16

**Authors:** Luisa Baader, Susan Schröpfer, Benye Liu, Stefanie Reim, Traud Winkelmann, Nils Orth, Jiem Krueger, Georg Guggenberger, Jens Boy, Jacqueline Kaldun, Eva Lehndorff, Nele Meyer, Ludger Beerhues, Kristin Hauschild, Henryk Flachowsky

**Affiliations:** Julius Kühn Institute (JKI) – Federal Research Centre for Cultivated Plants, Institute for Breeding Research on Fruit Crops, 01326 Dresden-Pillnitz, Germany; Julius Kühn Institute (JKI) – Federal Research Centre for Cultivated Plants, Institute for Breeding Research on Fruit Crops, 01326 Dresden-Pillnitz, Germany; Technische Universität Braunschweig, Institute of Pharmaceutical Biology, Braunschweig, Germany; Julius Kühn Institute (JKI) – Federal Research Centre for Cultivated Plants, Institute for Breeding Research on Fruit Crops, 01326 Dresden-Pillnitz, Germany; Institute of Plant Genetics, Leibniz University Hannover, 30419 Hannover, Germany; Institute of Plant Genetics, Leibniz University Hannover, 30419 Hannover, Germany; Institute of Earth System Sciences, Section Soil Science, Leibniz University Hannover, 30419 Hannover, Germany; Institute of Earth System Sciences, Section Soil Science, Leibniz University Hannover, 30419 Hannover, Germany; Institute of Earth System Sciences, Section Soil Science, Leibniz University Hannover, 30419 Hannover, Germany; Bayreuth Centre for Ecology and Environmental Research, University Bayreuth, 95448 Bayreuth, Germany; Bayreuth Centre for Ecology and Environmental Research, University Bayreuth, 95448 Bayreuth, Germany; Goethe University Frankfurt, Institute of Physical Geography, 60438 Frankfurt, Germany; Technische Universität Braunschweig, Institute of Pharmaceutical Biology, Braunschweig, Germany; Julius Kühn Institute (JKI) – Federal Research Centre for Cultivated Plants, Institute for Epidemiology and Pathogen Diagnostics, 38104 Braunschweig, Germany; Julius Kühn Institute (JKI) – Federal Research Centre for Cultivated Plants, Institute for Breeding Research on Fruit Crops, 01326 Dresden-Pillnitz, Germany

## Abstract

Apple replant disease (ARD) is a soil-borne disease that arises from replanting apple trees on land previously used for apple cultivation. There is interest in biomarkers that can reliably assess the severity of ARD by quantifying how strongly apple plants react to the disease in soils of different agro-environments. Thus far, transcriptomic studies of ARD-affected plants have examined only a few soils at a time, revealing that expression patterns vary among different soils. Here, we analyzed the expression of 90 candidate genes in the roots of apple plants (rootstock genotype ‘M.26’) grown in ARD-affected soils from 151 sites across Germany to test whether a consistent pattern of gene expression under ARD-conditions exists. Additionally, the expression of the candidate genes was analyzed in the leaves of apple plants grown in 18 different ARD-affected soils. Most of the genes (72) showed significantly upregulated expression in roots under ARD conditions, while only 11 showed significantly upregulated expression in leaves, suggesting that these genes play a significant role in the ARD reaction in roots but only a limited or no role in leaves. The candidate genes were evaluated for their potential as ARD biomarkers, defined by their consistently increased expression under ARD conditions across different soils and correlation with ARD severity. The accordingly selected ARD biomarker genes in roots include genes involved in phytoalexin biosynthesis, lignin metabolism, ethylene metabolism, cyanogenesis, detoxification, programmed-cell-death, and plant defense. These biomarkers have the potential to assess the severity of ARD and open up new possibilities for disease diagnosis.

## Introduction

Apple replant disease (ARD) is a soil-borne disease that arises from replanting apple trees on land previously used for apple cultivation and can persist in the soil for decades [3]. Affected plants typically exhibit stunted shoot growth and root damage, as well as reduced fruit yield and quality [4–6]. As a result, ARD poses a significant economic threat to orchards and tree nurseries worldwide, with estimated yield losses ranging from 20% to 50% [4, 5, 7].

Previous studies have shown that ARD is associated with harmful shifts in the soil biota community composition, and it is suspected that root exudates and the decomposition products of dead apple plant material induce these changes in the soil biome [3, 8, 9]. Many microorganisms have been described as being associated with ARD, including members of *Pythium* [4], *Rhizoctonia* [4], *Fusarium* [10], *Actinomycetes* [11], *Nectriaceae* [6, 11, 12], and *Streptomyces* [13]. At the same time, a decrease in taxa with presumable beneficial effects was recorded [14, 15]. Abiotic factors, such as soil texture, influence ARD severity, which often varies significantly, even within the same field [5, 16]. However, the replanting history of an orchard appears to play a bigger role than the soil characteristics [17].

Currently, there are few sustainable and effective countermeasures against ARD and the most common approaches, soil disinfection and crop rotation, are often impractical due to cost or environmental concerns [3]. Planting ARD-tolerant rootstocks appears to be the most reliable long-term method of mitigating the impact of the disease; however, the performance of commercially available tolerant rootstocks varies depending on the site [9]. Other promising ARD countermeasures, such as microbial inoculants, intercropping with *Tagetes*, and biofumigation with brassica seed meal, have produced inconsistent results as well [3, 5, 18]. Developing strategies for managing ARD requires a deeper understanding of the disease's underlying causes and the plant's response mechanisms [3, 19].

Transcriptomic studies of apple plants grown in ARD soil revealed the increased expression of genes associated with the biotic stress response [15, 19–24]. Among these genes, those involved in phytoalexin biosynthesis have been found to be especially overexpressed under ARD conditions. Recent studies have shown that biphenyl and dibenzofuran phytoalexins are induced in response to ARD and inhibit pathogenic, as well as commensal and beneficial, microorganisms [15, 17, 19, 20, 22, 25–27]. Other genes that have been shown to be differentially expressed under ARD conditions or after infection with ARD-associated pathogens are involved in the metabolism of phytohormones, flavonoids, lignin, and other secondary metabolites, as well as having functions related to signaling, detoxification, and pathogen defense processes [20, 21, 23, 24, 28–31]. However, these expression patterns vary between different soils and apple genotypes [17, 20, 22, 32, 33].

Fruit growers are interested to know whether and to what extent the soil on their site is affected by ARD, as this information could help them plan new orchards. Biomarkers that can detect and quantify how apple trees react to ARD could help evaluate ARD severity, management options and rootstock tolerance [22]. To reliably assess ARD severity, an evaluation system is needed that can be applied universally to soils from different agro-environments [19]. Thus far, transcriptomic studies of ARD-affected plants have examined only a few soils at a time [32]. To identify robust biomarker genes, experiments involving a large number of different ARD-affected soils are needed.

As a part of the joint BonaRes ORDIAmur project [34], 151 apple-replanted soils from different apple growing regions in Germany were utilized in an ARD biotest experiment as established by.

Yim *et al.* [14]. For this, ‘M.26’ apple plants sensitive to ARD were cultivated in untreated, potentially ARD-affected soils as well as in gamma-irradiated control samples of each soil. Experimental data from the various analyses, such as soil characteristics, plant growing parameters, as well as gene expression profiles, were collected by the project partners and are available in the BonaRes Repository [1, 2, 35].

The aim of this study is to identify universal biomarker genes for assessing the severity of ARD in different soil types. Based on previous studies, 90 candidate genes (CGs) will be selected that were either differentially expressed in apple plants under ARD conditions or shown to be involved in pathways induced by ARD-mediated stress. The expression of these CGs will be analyzed in roots and leaves derived from the large-scale experiment described above. Considering qPCR performance, differential gene expression, and their correlations with plant growth parameters, the large sample size will enable the evaluation of the CGs as ARD biomarkers with high statistical power.

## Results

### Selection of candidate biomarker genes

A total of 90 CGs were selected as potential biomarkers for ARD (Table S1). Eighty-six of these genes have previously been found to be differentially expressed in apple plants grown under ARD conditions [15, 19–24] or in apple plants infected by pathogens associated with ARD [28–31]. In addition, four putative phytoalexin biosynthesis genes, for which sequence information was provided by a collaboration partner (Liu *et al.,* unpublished), were included in the analysis. The main selection criterion for the CGs was the reported upregulation of their expression under ARD conditions. Additionally, genes showing higher upregulation in the ARD-tolerant genotype ‘Mxr.5’ compared to the ARD-susceptible genotype ‘M.9’ were preferably selected, as these genes are presumed to play a role in ARD tolerance [21]. Furthermore, genes with functions related to biotic stress response were preferentially chosen as well.

**Figure 1:**
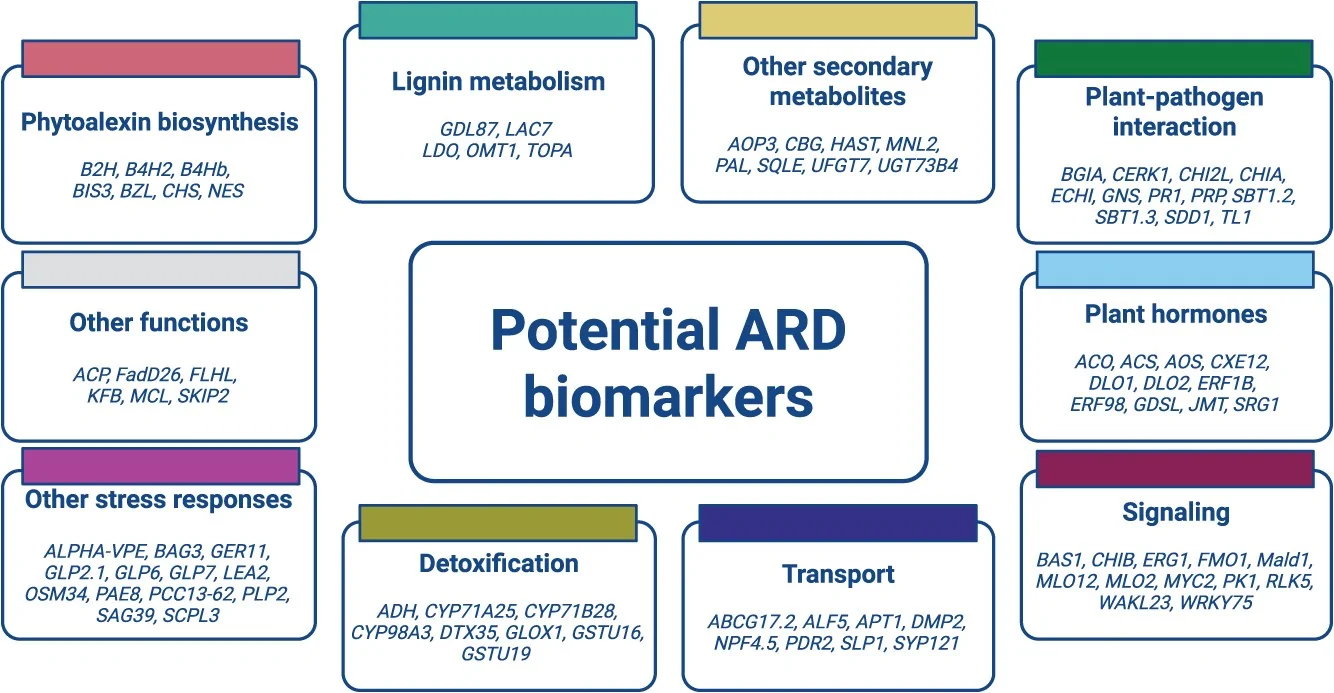
Functional classification of the selected potential ARD biomarkers. Based on literature, 90 CGs were selected that were shown to be differentially expressed in apple under ARD conditions, in apple plants infected by pathogens associated with ARD, or genes involved in pathways, which were described to be induced by ARD-mediated stress. These genes were classified into functional groups according to literature, UniProt and KEGG and their full names are listed in Table S1. Figure 1 was created with BioRender.com.

Based on the literature, UniProt [36] and KEGG [37] CGs were classified into functional groups (Fig. 1; Table S1). Seven of the selected genes play a role in the biosynthesis of phytoalexins, five genes in the metabolism of lignin, and eight genes in the metabolism of other secondary metabolites. These include the synthesis of phenylpropanoids, flavonoids, sesquiterpenoids and triterpenoids, cyanides, and glucosinolates. Twelve CGs have functions related to plant–pathogen interactions, including genes involved in the recognition and response to pathogens. Out of the eleven genes playing a role in plant hormone metabolism and signaling, eight are related to ethylene, two to jasmonate, and one to auxin. Twelve CGs have functions related to signaling, and eight each to transport and detoxification. The 13 genes classified as playing a role in other stress responses include genes involved in programmed cell death, as well as genes related to biotic and abiotic stress response. The remaining six genes have other functions, such as being involved in the metabolism of lipids or protein processing.

### Establishing gene-specific primers for high-throughput real-time PCR

For 21 of the CGs, qPCR primer sequences were already available, while for the other 69 genes primers had to be established (Table S1). A total of 79 primer pairs were designed with NCBI Primerblast and tested for specificity *in silico* by blasting against the *Malus domestica* transcriptome (taxid:3750). In order to rule out qualitative errors in advance, the new primer pairs were tested in an establishing qPCR prior to the high-throughput qPCR. Only the primers that showed the expected amplification product size in gel electrophoresis, exhibited one specific melt-curve peak, and demonstrated sufficient amplification efficiency were selected. Any primer pairs that failed the quality check were discarded and redesigned.

### Quality parameters for expression of reference and CGs

**Figure 2:**
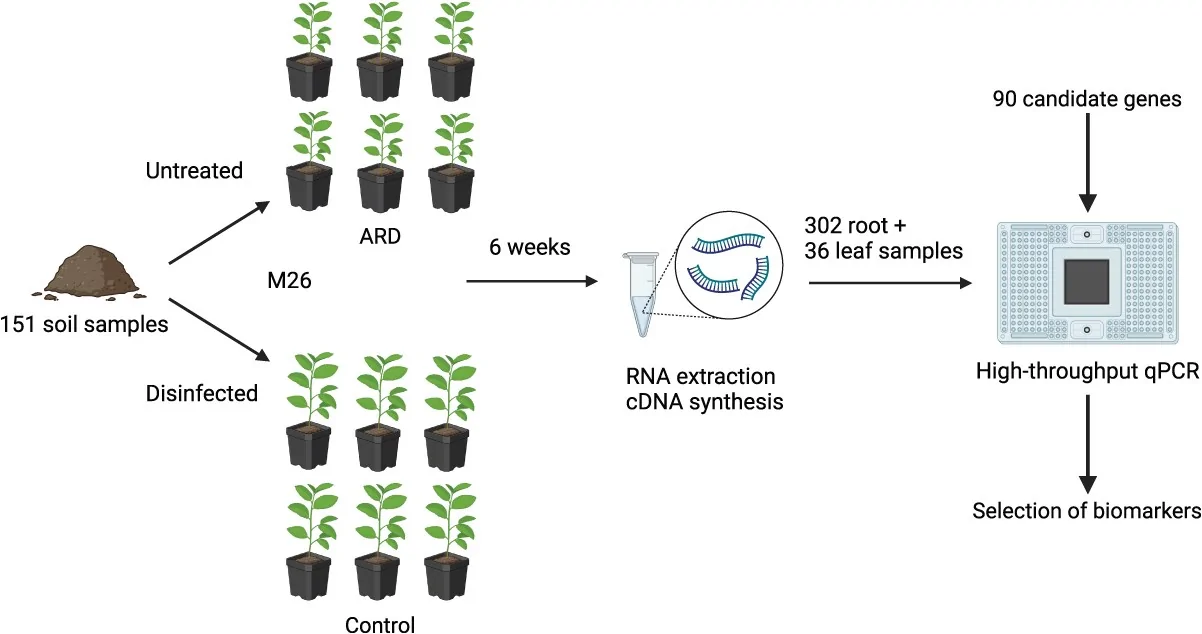
Experimental design. A total of 151 soil samples were collected from different apple growing regions across Germany that were affected by ARD. ‘M.26’ apple plants were grown in a greenhouse biotest for six weeks under ARD conditions using an untreated soil sample. A soil sample that was disinfected using gamma-irradiation served as corresponding control. Samples were taken from the roots of six plants from both the ARD and the control variants in each soil type. In addition, leaves from six plants in the ARD and control variants were sampled for 18 soils. Per soil and treatment, the samples were pooled, and total RNA was extracted and reverse-transcribed into cDNA. Gene expression analysis of the 90 CGs was performed by qPCR using the BioMark HD high-throughput system and based on the results, the most promising potential biomarker genes were selected. Figure 2 was created with BioRender.com.

To analyze the CGs' expression in response to ARD conditions in root and leaf tissue, plants of the ‘M.26’ rootstock were grown for 6 weeks in 151 different ARD-affected soil samples (Fig. 2). In accordance with the biotest established by Yim *et al.* [14], samples of each soil that were disinfected through gamma-irradiation served as ARD-unaffected controls. The origins and characteristics of the soils and the growth difference of the plants grown in ARD and control soil are available in the BonaRes Repository [1, 35]. The shoot fresh mass reduction in ARD soils compared to their respective control soil ranged from −74.5% to 77.9% and was significantly reduced in 103 soils. The shoot dry mass reduction (−76.3% to +72.3%) was significant in 100 ARD soils. As an example, Fig. S1 illustrates the growth reduction of plants grown in the untreated and disinfected variants of one soil.

The expression analysis of the 90 CGs (Table S1) and 5 reference genes (Table S2) was performed by qPCR using the BioMark HD high-throughput system. The stability of the reference genes was analyzed with RefFinder, a tool for evaluating the stability of reference genes that combines the programs BestKeeper, the comparative ΔCt method, geNorm, and NormFinder [38]. The comprehensive stability ranking was as follows, from most to least stable: *EF1b, ACT7, EF1a, TUBB, UBE210* (Table S3). Because *UBE210* consistently ranked last, it was excluded from further analysis and only *ACT7, EF1a, EF1b*, and *TUBB* were used as reference genes.

The qPCR showed efficiencies between 98% and 100% for 70 primer pairs and efficiencies above 90% for 15 further primer pairs (Table S4). The qPCR with the *SRG1* primers exhibited the lowest efficiency at 86%, and for the primer pairs *FMO1, GER11, GLP7*, and *JMT,* no amplification efficiencies could be calculated, as the standard samples failed the quality check of the Fluidigm software.

### Expression of the CGs in response to ARD in roots

A total of 58 740 qPCR reactions were performed using the high-throughput system. Of these, 5239 were excluded from analysis due to technical issues. In order to calculate the relative expression (fold change values) of each gene per soil sample, both the ARD sample and the corresponding control sample had to pass the technical quality check. Therefore, the number of samples included in the relative expression analysis differs among the genes (Table S4). For the majority of the genes (58), samples from plants grown on at least 140 soils were used in the analysis. The analysis used samples from over 100 soils for 21 of the genes and over 40 soils samples for 10 genes. For the *GLP7* gene, the relative expression could be calculated for only three samples.

**Figure 3:**
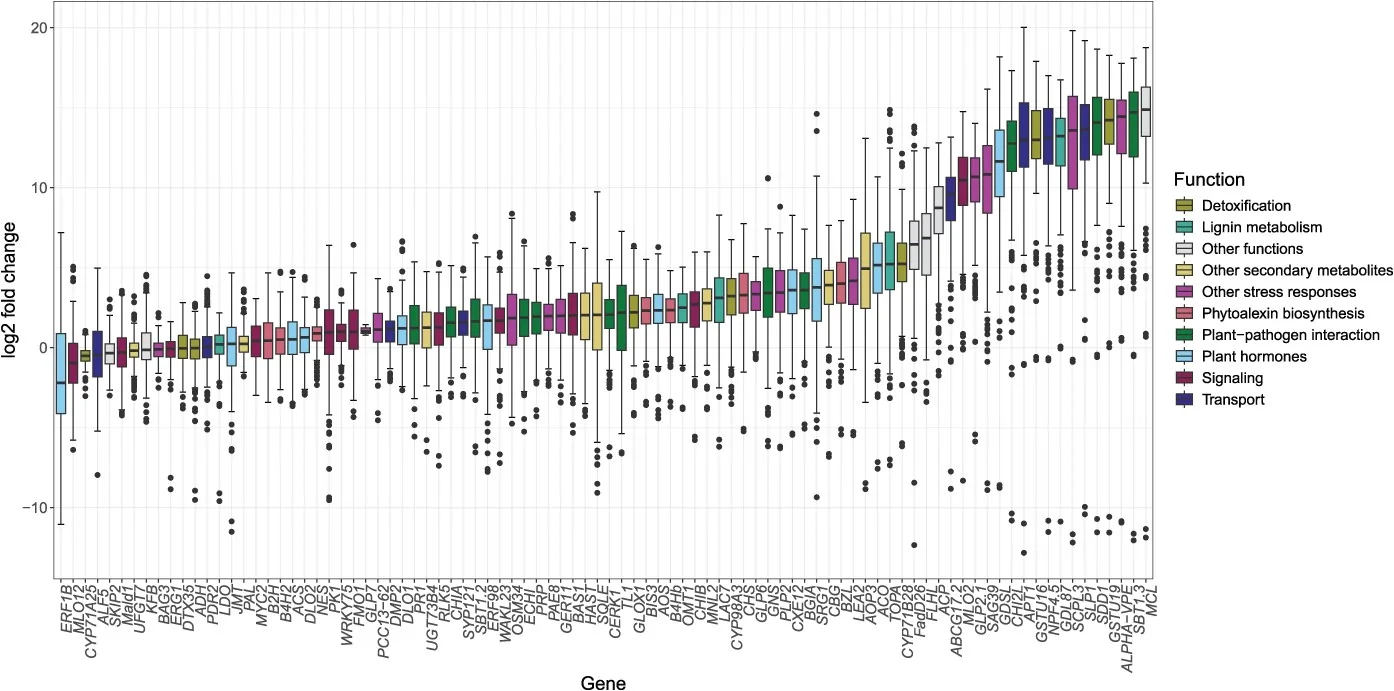
Differential gene expression in apple roots under ARD conditions compared to control conditions. The diagram shows the differential expression of 90 CGs (Table S1) in the roots of apple plants in response to ARD conditions. The log_2_ fold change (ARD-affected soil/disinfected control soil) is depicted as a boxplot, calculated with data obtained from up to 151 different soils.

Nearly all CGs have been documented in the literature as being upregulated under ARD conditions. However, a wide variation in their differential expression was observed in this study (Fig. 3), with the fold change values of the individual samples ranging from 0.0001 to 1 064 865 (log₂ fold-change (log_2_FC): −12.8 to 20.0). The gene expression data for each soil sample are available in the BonaRes Repository [2]. Seven genes showed lower expression on average under ARD conditions compared to control conditions (*CYP71A25, ERF1B, ERG1, Mald1, MLO12, SKIP2, UFGT7*), while the expression of 11 genes was not significantly changed on ARD soil compared to the control (Table S4). The remaining 72 genes were, on average of all soils, significantly upregulated under ARD conditions (*P* < 0.05). Of these, 57 genes exhibited average fold change values above 4.5 (log_2_FC: 2.2), seven genes exhibited average fold changes between 100 and 1000 (log_2_FC: 6.6 to 10.0), and 17 genes exhibited average fold changes above 1000. Figure S2 shows the relative expression values of three example genes for each soil sample, illustrating how the relative expression of each gene varies between the soils.

### Expression of novel postulated phytoalexin biosynthesis genes was upregulated under ARD conditions

For the first time, this study analyzed the expression of the genes *B2H*, *B4H2*, *BLZ,* and *NES*, which are assumed to be involved in phytoalexin biosynthesis (Liu *et al.,* unpublished), in apple roots under ARD conditions. The expression of the *B2H*, *B4H2*, and *BLZ* genes were found to be significantly upregulated in ARD-affected roots (*P* < 0.05; Table S4). The *NES* gene expression also showed significant upregulation; however, the qPCR with the *NES* primers exhibited nonspecific amplification in several samples.

### Evaluation of the CGs for their suitability as biomarkers for ARD in roots

The present study primarily used root samples for the evaluation of the CGs as biomarkers for ARD, because the roots are the organs directly exposed to ARD-affected soil, and the previous transcription analyses have mainly focused on root tissue [15, 19–24, 28–31]. For the evaluation, several factors were taken into account, and each gene was assessed individually. The results of the evaluation are recorded in Table S6.

The first selection criterion was the qPCR performance of the gene-specific primer pair, which encompasses both amplification efficiency and specificity (Table S6, PCR quality), as well as the number of soil samples that passed the Fluidigm software quality check (Table S6, n soils). For instance, the PCR performed with *GLP7-*specific primers was disregarded because only three samples passed the qPCR quality check and *ALF5* was excluded because the melt curve analysis indicated nonspecific amplification. Additionally, the strength of expression was taken into consideration, as higher expression levels make detection by qPCR more robust (Table S6, Ct). A total of 24 genes were deselected due to their poor qPCR performance (Table S6, assessment: PCR issues).

The second selection criterion was the differential expression of each gene under ARD conditions. For this, the mean fold change value for all soil samples was evaluated (Table S6, FC, log_2_FC), and the significance of the upregulation under ARD conditions was calculated based on the calibrated normalized relative quantification of gene expression (Table S6, difference ARD/control). Sixteen genes were deselected due to a lack of upregulated expression, and an additional eight genes were deselected due to low fold changes (Table S6, assessment: low fold change). Furthermore, the consistency of differential expression across the different soils was assessed for each gene, as illustrated exemplarily in Fig. S3 for two genes. Both genes exhibited significantly upregulated expression under ARD conditions and similar mean fold changes (Table S6); however, the expression of *BGIA* was induced much more consistently across the different soils than that of *OSM34* (Fig. S3). A total of 17 genes were deselected due to inconsistent upregulation of expression (Table S6, assessment: inconsistent upregulation).

The third criterion was the correlation of the differential gene expression with the growth difference of plants grown in ARD and control soil (Table S7). These growth parameters encompass the shoot dry mass reduction, shoot fresh mass reduction and root fresh mass reduction, and serve as indicators of ARD severity [1, 39]. Overall, genes with moderate fold change values showed stronger correlations than those with very high values. A total of 13 genes were deselected due to lack of correlation with the growth parameters (Table S6, assessment: lack of growth correlation).

**Figure 4:**
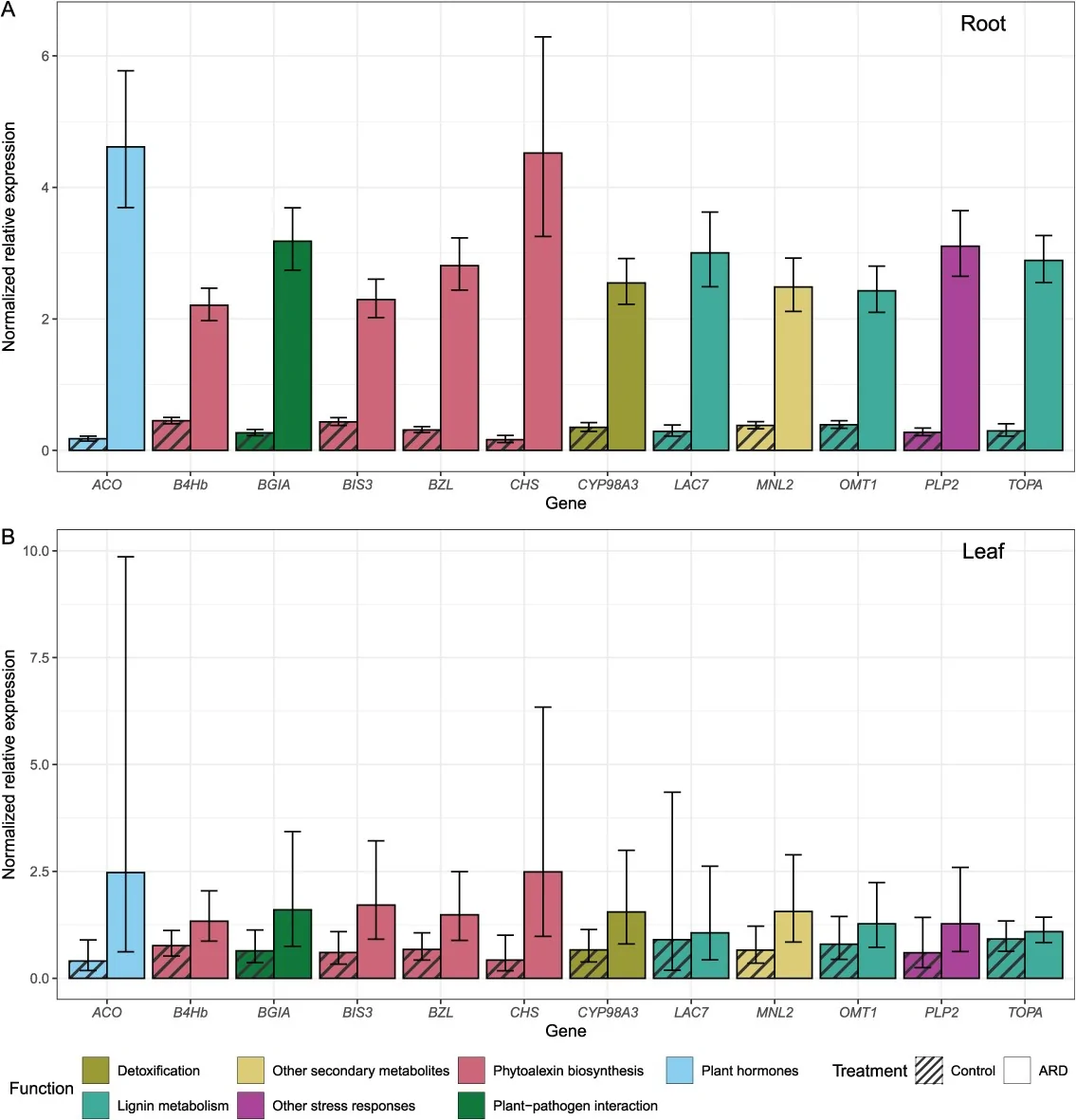
Normalized relative expression of the suggested ARD biomarkers. The expression of the 12 genes classified as the most promising ARD biomarkers was analyzed by qPCR in the roots (A) and leaves (B) of apple plants grown on up to 151 (roots) or 18 (leaves) ARD-affected soils and disinfected control samples of each soil. The normalized relative expression values are the averages for all soils, and the 95% confidence interval is represented as error bar. (A) The expression of the genes was significantly higher in the roots of ARD-affected plants compared to the control (*P* < 0.001). (B) Only the expression of *CHS* was significantly higher in the leaves of ARD-affected plants compared to the control (*P* < 0.05).

Based on the evaluation, the qPCR results for 12 genes that were rated positively in all criteria were designated as the most promising potential ARD biomarkers (category I, Table 1). For 35 genes, the evaluation was positive for the majority of the criteria, resulting in the classification as potential ARD biomarkers (category II, Table S6). Lastly, 43 genes were deemed not suited as ARD biomarkers based on the results of the performed qPCRs (category III, Table S6). Category I includes four phytoalexin biosynthesis genes: *B4Hb, BIS3, BZL*, and *CHS*. *CHS* is also involved in flavonoid biosynthesis. Three genes involved in lignin metabolism, *LAC7*, *OMT1*, and *TOPA*, were also classified as category I, as were the cyanogenesis related gene *MNL2*, the ethylene biosynthesis gene *ACO*, the plant defense related gene *BGIA*, the detoxification related gene *CYP98A3*, and the cell death related gene *PLP2*. Figure 4A shows that the normalized relative expression of the category I genes was significantly higher (P < 0.001) in the roots of ARD-affected plants compared to roots of plants grown in the control soils.

**Table 1:** CGs rated most promising for their suitability as biomarkers for ARD in roots.

**Gene**	**Ct**	**FC**	**Log** _**2**_ **FC**	**Difference ARD/Control**		**PCR quality**	**n soils**	**Correlation SDM_red**	
*ACO*	10.08	76.98	4.70	0.00E+00	***	Good	148	0.310	***
*B4Hb*	5.54	6.14	2.21	0.00E+00	***	Good	129	0.339	***
*BGIA*	5.99	19.25	3.47	0.00E+00	***	Good	144	0.496	***
*BIS3*	4.24	6.29	2.18	0.00E+00	***	Good	130	0.324	***
*BZL*	10.54	31.36	3.89	0.00E+00	***	Good	149	0.205	***
*CHS*	7.63	18.70	3.24	5.43E-16	***	Good	149	0.225	***
*CYP98A3*	9.99	15.22	2.98	0.00E+00	***	Good	149	0.386	***
*LAC7*	14.47	20.80	2.92	0.00E+00	***	Good	149	0.256	***
*MNL2*	6.09	10.06	2.59	0.00E+00	***	Good	138	0.347	***
*OMT1*	5.74	7.55	2.35	0.00E+00	***	Good	138	0.414	***
*PLP2*	13.32	25.28	3.44	0.00E+00	***	Good	148	0.411	***
*TOPA*	10.48	612.81	5.40	0.00E+00	***	Good	147	0.244	***

The CGs were evaluated for their potential to serve as biomarkers for ARD. This table shows the most promising potential ARD biomarkers (category I), the evaluation of the other genes can be found in Table S6. For each gene the table shows the mean Ct value of the ARD samples (Ct), the mean fold change (FC), and log_2_ fold change (log_2_ FC), the *P* value and significance level of a paired Wilcoxon signed rank test of difference in gene expression between the ARD and control samples (difference ARD/Control), the qPCR performance of the gene specific primer pair (PCR quality), the number of successfully analyzed soil samples (n soils), and the Spearman correlation coefficient and significance level of gene expression with shoot dry mass reduction (SDM_red). Significance level: *** *P* < 0.001.

**Table 2:** CGs rated most promising for their suitability as biomarkers for ARD in leaves.

**Gene**	**Ct**	**FC**	**Log** _**2**_ **FC**	**Difference ARD/Control**		**PCR quality**	**n soils**	**Correlation SDM_red**	
*B2H*	11.01	5.97	1.43	3.94E-02	*	Good	18	0.534	**
*CHS*	12.56	7.20	1.73	1.97E-02	*	Good	18	0.296	*
*OSM34*	8.49	5.10	1.59	2.18E-02	*	Good	18	0.532	**
*PCC13–62*	15.86	4.49	1.62	9.83E-03	**	Good	18	0.686	***

The CGs were evaluated for their potential to serve as biomarkers for ARD. This table shows the most promising potential ARD biomarkers (category I). The evaluation of the other genes can be found in Table S8. For each gene the table shows the mean Ct value of the ARD samples (Ct), the mean fold change (FC) and log_2_ fold change (log_2_ FC), the result of a paired Wilcoxon signed rank test of difference in gene expression between the ARD and control samples (difference ARD/Control), the qPCR performance of the gene specific primer pair (PCR quality), the number of successfully analyzed soil samples (n soils), and the Spearman correlation coefficient and significance level of gene expression with shoot dry mass reduction (SDM_red). Significance levels: *** *P* < 0.001, ** *P* < 0.01, * *P* < 0.05.

### Expression of the CGs in leaves in response to ARD

**Figure 5:**
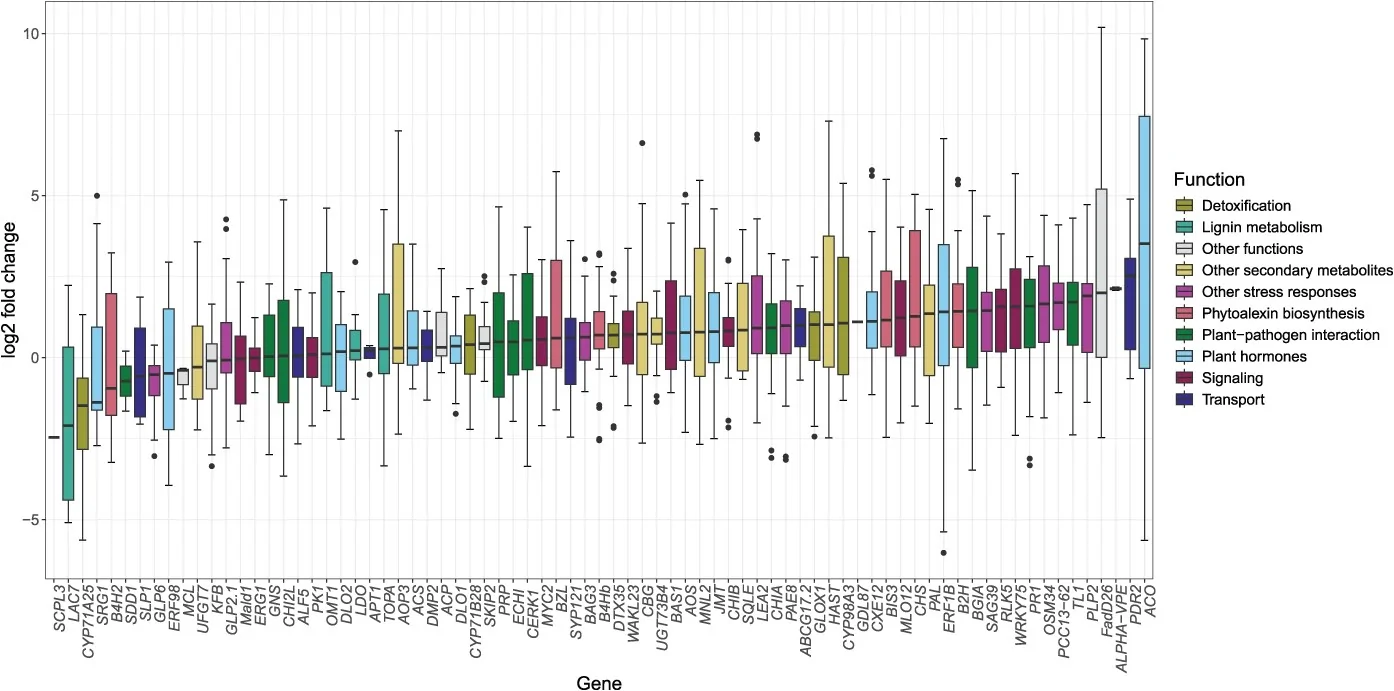
Differential gene expression under ARD conditions in leaves. The diagram shows the differential expression (log_2_ fold change values) of 77 CGs (Table S1) in the leaves of apple plants in response to ARD conditions. The log_2_ fold change values (ARD-affected soils/disinfected control soils) are depicted as a boxplot, calculated with data obtained from up to 18 different soils. The expression of the remaining 13 CGs in leaves was too low to obtain usable data.

Since taking leaf samples is much more convenient and feasible than taking root samples, it would be preferable to use leaf material to analyze biomarker gene expression. Thus, the expression of the 90 CGs was analyzed in the leaves of apple plants grown in a subset of 18 different ARD-affected soils and disinfected control samples of each soil (Fig. 5). A total of 6840 qPCR reactions were performed using the high-throughput system. Of those, 1718 were excluded from the analysis due to technical issues, proportionally three times more than recorded for the root samples (see above). The expression of 13 genes in leaves was too low to obtain usable data (Table S5). For 50 genes, samples from plants grown in 18 soils were used in the analysis; for 14 genes, samples from over 10 soils were used; and for 13 genes, samples from fewer than 10 soils were used. The differential expression of the CGs under ARD conditions exhibited lower fold changes in leaves than in roots, as the fold change values for individual samples ranged from 0.015 to 171.43 (log_2_FC −6.02 to 10.19). The gene expression data for each soil sample is available in the BonaRes Repository [2].

Overall, the significance test of the differential gene expression showed higher *P* values in the leaf samples compared to the root samples (Tables S4 and S5). A total of 11 genes exhibited significantly upregulated expression in leaves under ARD conditions, with six genes exhibiting average fold change values greater than 4.5 (log₂FC: 2.2; Table S5). Of the 12 genes identified as the most promising biomarkers in roots, only the *CHS* gene was found to be significantly overexpressed (*P* < 0.05) in leaves as well (Fig. 4B). Interestingly, the *CYP71A25* and *GLP6* genes showed, on average, higher levels of expression in the control compared to the ARD samples. The remaining 64 genes did not show a significant change in expression in ARD soil in comparison with the control.

### Evaluation of the CGs for their suitability as biomarkers for ARD in leaves

**Figure 6:**
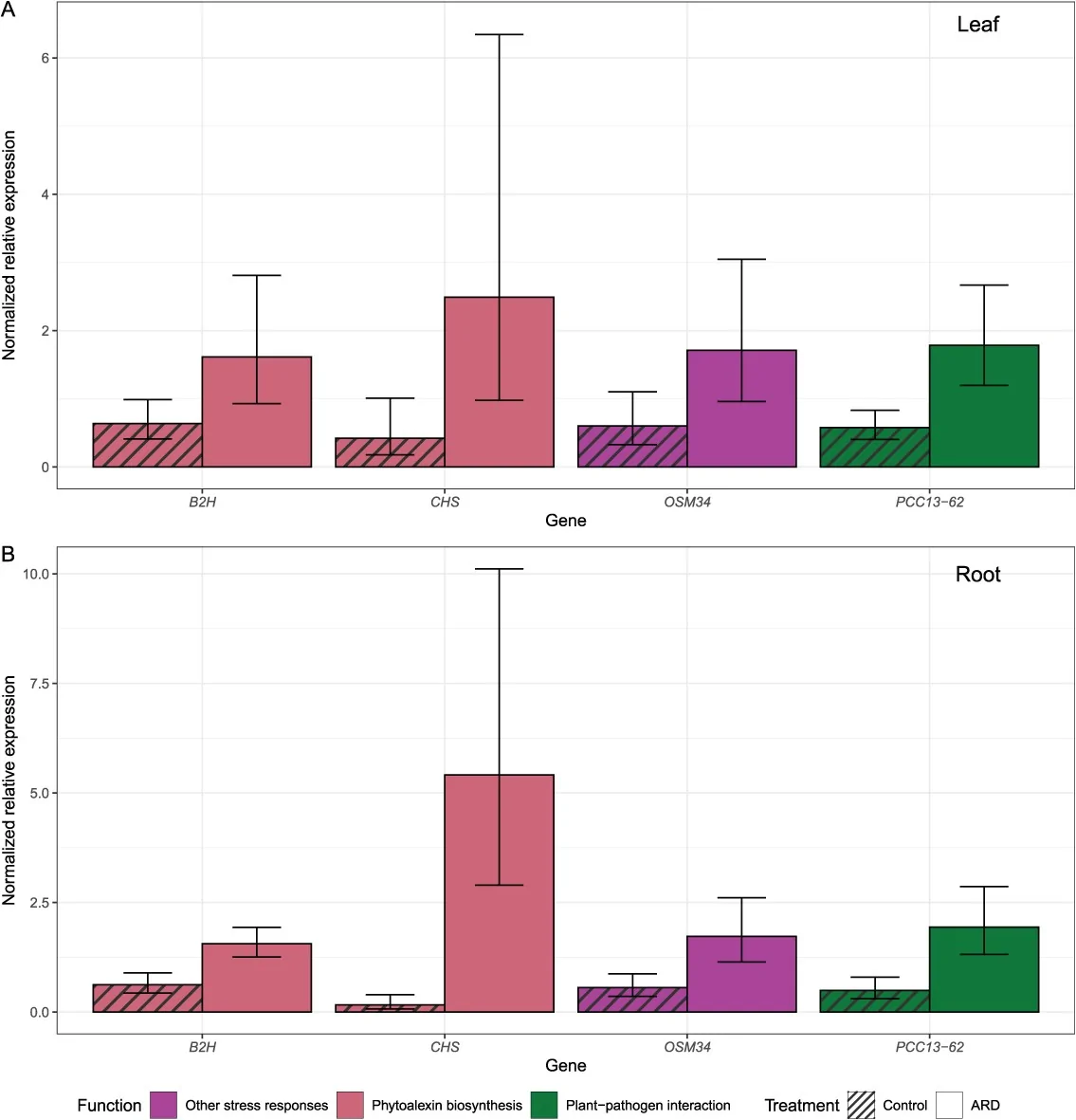
Normalized relative expression of most promising potential ARD biomarkers in leaves. The expression of the four genes classified as the most promising ARD biomarkers in leaves was analyzed by qPCR in the leaves (A) and roots (B) of apple plants grown on up to 18 ARD-affected soils and disinfected control samples of each soil. The normalized relative expression values are the averages for all soils, and the 95% confidence interval is represented as error bar. The expression of the genes was significantly higher in ARD-affected plants compared to the control (*P* < 0.05).

As with the gene expression in roots, the CGs were evaluated for their potential of being used as biomarkers in leaves and classified into three categories based on their qPCR performance, their differential expression, and their correlation with the growth parameters (Table S8). Based on the evaluation, four genes were classified as the most promising potential ARD biomarkers (category I, Table 2), 17 genes were classified as potentially suitable ARD biomarkers (category II, Table S8), and the qPCR results revealed 69 genes to be not suited as ARD biomarkers (category III, Table S8). The category I genes include the phytoalexin and flavonoid biosynthesis gene *CHS*, which was identified as one of the most promising potential biomarker genes in roots as well*.* A second putative phytoalexin gene, *B2H*, was also classified as category I gene, as were the stress response–related gene *PCC13-62* and the pathogen defense response gene *OSM34.*  Figure 6 shows that the normalized relative expression of the category I genes was significantly higher (*P* < 0.05) in ARD-affected plants compared to the control.

## Discussion

### The majority of CGs was upregulated in roots under ARD conditions

In order to reliably assess the severity of ARD in a soil, there is interest in biomarkers that can universally detect and quantify the ARD response of plants, regardless of the soil's agro-environmental origin [22, 23]. This study analyzed the expression of 90 CGs in the roots of ‘M.26’ plants grown in ARD-affected soils from 151 different sites across Germany. To compare apple plants grown in ARD soils to those grown in non-ARD soils with the same properties, half of each soil sample was left untreated, while the other half was disinfected using gamma-irradiation. This method has been used in many previous studies [14, 17, 19–24, 32, 40] and has the advantage of reversing ARD through disinfection while preserving most of the chemical and physical soil properties.

Overall, a wide variation in the degree of upregulation of gene expression was observed (Fig. 3), with some genes exhibiting very high fold changes of over 1000 (log_2_FC: 10.0). These very large fold change values were caused by extremely low expression in the control samples and higher expression in the ARD samples. The expression of the majority of the genes (72) was significantly upregulated under ARD conditions (Table S4). However, the remaining 18 genes were not significantly upregulated in roots from ARD-affected soils compared to roots from control soils. This finding was unexpected, as nearly all CGs (86) were selected for their reported overexpression under ARD conditions. For example, *ERF1B,* an ethylene-related gene previously recommended as an ARD biomarker [20, 22, 23], showed a wide variance in differential expression among different soils in this study. *ERF1B*-fold changes ranged from 0.0004 to 114, and *ERF1B* expression was upregulated in 39 soils but downregulated in 74 soils [2]. These results align with those of Orth *et al.* [17], who found that *ERF1B* expression was only upregulated in one of the five tested ARD-affected soils. This demonstrates the significant influence that soil has on the plants' molecular reactions to ARD due to the different abiotic and biotic soil characteristics, as well as the replant histories [17, 20, 22]. Therefore, it is crucial for the identification of robust biomarker genes to analyze their expression in a large number of different ARD-affected soils.

Another reason for the lack of agreement with the previous literature of the expression data for some of the genes could be that the genes were analyzed in different apple genotypes [20, 21, 32, 33]. Additionally, some genes were shown to be upregulated in previous RNA-sequencing analyses [21]; however, their expression may be too low for robust detection by the performed qPCR assays (e.g. *FMO1, GER11,* and *GLP7*). Furthermore, a technical consideration has to be taken into account, because high-throughput qPCR using the BioMark HD system was susceptible to technical problems, such as air bubbles in the reaction chambers. The strict quality control measures excluded samples with these errors. Finally, different primers may produce better results for some genes and could be designed and tested in future studies.

In this study, the expression of nine genes was described as being upregulated in apple plants grown in ARD affected soil for the first time. These include the putative phytoalexin biosynthesis genes *B2H*, *B4H2*, *BZL*, and *NES*, confirming the important role of phytoalexins in the ARD plant response. Previous studies have shown the induction of phytoalexin-related genes, as well as the correlation of their expression with phytoalexin production and ARD symptom severity [17, 20, 22, 23, 26]. Furthermore, the expression of CGs, which were previously only described after infection with ARD-associated pathogens, was shown to be upregulated in in this study. These comprise *ACS*, *CBG*, *CERK1*, *SQLE* [29, 30], and *ERF98* [31].

### Promising potential ARD biomarkers in roots have various functions in the ARD response

The twelve genes evaluated as the most promising potential ARD biomarkers in roots (category I, Table 1) were significantly and consistently upregulated under ARD conditions compared to control conditions across different soils. Their expression correlated with ARD severity (Table S6), which was measured by the reduction of plant growth [39].

The potential ARD biomarkers include *BIS3* and *B4Hb*, which have been previously recommended as biomarkers as well [15, 19–23, 32]. These genes are involved in biphenyl formation in the phytoalexin biosynthetic pathway [41, 42] and their expression has been shown to correlate with phytoalexin formation and ARD symptoms [17, 20, 22, 32]. Furthermore, additional genes from category I, *BZL* and *CHS*, are also related to phytoalexin formation. Lastly, the *ACO* gene was also classified as category I and is involved in ethylene biosynthesis, which can induce phytoalexins of the phenylpropanoid pathway [43]. This underpins the importance of the phytoalexin pathways during the ARD response of the plant. It is known that apple roots exude phytoalexins into the soil in a compound-specific percentage, where they play a role in shaping the soil microbiome through their antimicrobial activities, which might influence ARD severity [17, 44]. However, the overall impact of phytoalexins on ARD development is not yet fully understood, as they can cause a decrease of both pathogenic and beneficial microorganisms in the soil. The induction of phytoalexins in response to ARD is genotype-specific, and the production of individual phytoalexins may influence the ARD susceptibility of different genotypes [27]. Weiß *et al.* [19] hypothesized that the phytoalexin defense response of the ARD-susceptible genotype ‘M.26’, which was used in this study, is ineffective against ARD and that the roots might even be damaged by the high concentration of phytoalexins.

Further genes classified in category I include the lignin biosynthesis genes *LAC7*, *OMT1*, and *TOPA*. Reim *et al.* [21] proposed that increased cell wall stabilization through lignin biosynthesis reduces the negative effects of ARD on plants, which were also evident in term of disruptions of the outer root cell layers [14]. In this study, all analyzed lignin metabolism genes were significantly upregulated under ARD conditions, which emphasizes their role in the ARD response. Interestingly, *LAC7* and other laccase genes have shown increased expression after infection with the ARD-associated pathogen *Pythium ultimum* [30]*.* Zhu *et al.* [45] discovered that *P. ultimum* infection leads to the downregulation of a laccase-targeting miRNA, particularly in tolerant genotypes. The downregulation of this miRNA could lead to faster and stronger cell wall stabilization through increased laccase activity and thereby contribute to tolerance against the pathogen. Corroborating this finding, a QTL for *P. ultimum* tolerance has been identified near the putative location of this miRNA [46].

Another gene in category I is *CYP98A3*, which belongs to the cytochrome P450 superfamily. This family is involved in the detoxification of reactive oxygen species and bioactive compounds [47]. The category I gene *MNL2* has its function in cyanogenesis, which can play a role in the defense against fungi [48, 49]. The expression of other cyanogenesis genes has been found to be induced after infection with *P. ultimum* [30], and the cyanogenic gene *CBG* was found upregulated in this study as well. Another gene in category I is *PLP2*, which plays a role in programmed cell death and may restrict pathogen growth [50]. Lastly, the gene *BGIA* was classified in category I. In *Arabidopsis,* a *BGIA* homolog was found to serve a function in plant defense and response to wounding [51].

All things considered, we suggest that the expression response of category I genes could be used as a diagnostic tool for detecting and quantifying ARD across a wide range of soils under controlled test conditions. However, other soil-independent factors may influence the expression of the proposed ARD biomarker genes. These factors should be controlled for or investigated in future studies, e.g. analyzing the expression under both abiotic and biotic stress conditions. Thus far, only *BIS3* and *B4Hb* have been examined in apple plants under abiotic stress conditions, where neither showed an upregulation response [22]. Furthermore, the ARD biomarkers' expression should also be investigated in other rootstock genotypes and at earlier time points after planting.

### The ARD response gene expression pattern differs between roots and leaves

Since leaf samples are more convenient to take than root samples, it would be preferable to use leaf material to analyze biomarker gene expression. Thus, the expression of the CGs was also analyzed in the leaves (Fig. 5). Overall, the genes showed less significant differential expression in the leaf samples than in the root samples (Table S5), as expected, since most of the genes have previously only been described in roots. Several of the genes were barely expressed in the leaves. These results align with those of Weiß and Winkelmann [24] and Reim *et al.* [20], who also found lower gene expression and upregulation in leaves. However, the genes that were previously identified as being upregulated in leaves upon growth in ARD-affected soils were not significantly upregulated in this study, which may be due to the use of different soils.

Of the genes identified as the most promising biomarkers in roots, only the phytoalexin and flavonoid biosynthesis gene *CHS* was also classified as category I in leaves. A second putative phytoalexin gene, *B2H*, was also classified as a category I gene. Weiß and Winkelmann [24] also found the induced expression of many phytoalexin genes in leaves as well, suggesting a delayed systemic response to ARD. Other category I genes in leaves include the stress-responsive antifungal gene *OSM23,* which has been found to be overexpressed in *Arabidopsis* infected with a fungal pathogen [52]. The *PCC13-6* gene was classified as category I and plays a role in the abiotic water stress response [53], pointing to potential water stress in the leaves of ARD-affected plants due to a damaged root system.

Given the smaller expression changes observed in leaves, it is questionable whether the sensitivity of the proposed biomarker genes is sufficient for a reliable assessment of the ARD response, or whether only strong ARD contamination can be diagnosed. This issue should be addressed in subsequent studies.

## Conclusion

In conclusion, this study identified genes that were consistently upregulated under ARD conditions across many different soils. The results indicate that these genes play a significant role in the ARD reaction in roots, but only to a limited extent in leaves. Potential ARD biomarkers that could reliably assess ARD severity were selected. However, future studies are needed to analyze the selected genes’ specificity to ARD and their performance in other apple genotypes.

## Materials and methods

### Soil and plant material

Surface soil (0–30 cm) was collected from 151 different sites with apple cultivation history in Germany. Half of each soil was disinfected by gamma-irradiation (>10 kGy). Successful disinfection was validated via plating of soil suspensions (1 g in 9 ml 0.85% NaCl) onto R2A agar (Carl Roth, Germany) with 100 mg∙L^−1^ cycloheximide or 1/3 potato dextrose agar (PDA, Carl Roth, Germany) with 100 mg∙L^−1^ penicillin, 10 mg∙L^−1^ tetracycline, 50 mg∙L^−1^ streptomycin. Colony forming units (CFUs) were counted after inoculation for 48 hours (bacteria, R2A) or 7 days (fungi, PDA) at room temperature in the dark.

*In vitro* propagated and rooted young plantlets of the rootstock ‘M.26’ were acclimatized for four weeks in a peat substrate as described by Rohr *et al.* [40]. The biotest was carried out in nine sets of up to 18 soils between 13.07.2022 and 20.08.2023. Each set included one variant with peat substrate to normalize possible growth differences among the sets. Each of the 151 soils used as untreated ARD-affected soil (ARD) and gamma-irradiated soil (control) was first supplemented with 2 g L^−1^ of the slow-release fertilizer Osmocote exact 3–4 M (N–P_2_O_5_–K_2_O–MgO: 16–9–12–2, ICL Deutschland, Germany) and per soil variant eight pots (600 ml each) were prepared with one ‘M.26’ plantlet per pot. The pots were placed randomly onto four tables in a climate chamber and cultivated for six weeks (19.9 ± 2.6°C, 61.9 ± 7% relative humidity, 16/8 hours day/night cycle with SON-T AGRO 400 W lamps (Philips, Netherlands)). Irrigation with tap water was done manually on a daily basis. For evaluation of plant growth and sampling, the two plants with the smallest and largest shoot length were excluded and the remaining six plants per soil variant were used to collect growth data and samples. On the day of sampling, the roots were carefully washed with tap water. Per soil and treatment, 100 mg roots and the first fully expanded leaf of each of the six selected plants were pooled (roots and leaves separately) and immediately frozen in liquid nitrogen. The samples were stored at −80°C until RNA isolation.

### RNA isolation and cDNA synthesis

Root and leaf pooled samples were homogenized with a Mixer Mill MM400 (Retsch, Germany) at 25 Hz for 2 minutes while cooled by liquid nitrogen. With the InviTrap Spin Plant RNA Mini Kit (Invitek Molecular, Germany), total RNA was isolated according to the manufacturer's instructions with RP lysis buffer and the RNA was eluded in 60 μl elution buffer R. Genomic DNA was removed with DNase I (Thermo Fisher Scientific, USA) following the manufacturer's instructions. RNA concentrations were measured using the Nanodrop One (Thermo Fisher Scientific, USA). The cDNA synthesis was performed with the RevertAid First Strand cDNA Synthesis Kit (Thermo Fisher Scientific, USA) using oligo dT primers and 1 μg RNA as template. The cDNA synthesis was verified in a PCR with *EF1* primers.

(5′-ATTGTGGTCATTGGYCAYGT-3′/5′-CCAATCTTGTAVACATCCTG-3′) using 1 μl cDNA and genomic DNA as control [54]. The amplified PCR products from the cDNA and the genomic DNA have different fragment sizes (707/905 bp) and no product would be generated with only RNA as template. Verified cDNA samples were stored at −20°C until further use.

### Primer selection and qPCR validation

A total of 90 CGs were selected as potential biomarkers for ARD (Table S1). For 61 of these genes, new qPCR primers were designed with NCBI Primer Blast and tested *in silico* for specificity by blasting against the *Malus domestica* transcriptome (taxid:3750). The primer pairs were then tested for efficiency and specificity by qPCR using the BioRad CFX96 Real-Time System (Bio-Rad Laboratories, USA). The qPCR was performed with the Maxima SYBR Green master mix (Thermo Fisher Scientific, USA) using 1 μl ‘M.26’ apple cDNA diluted 1:10, 1:100, and 1:1000 as template and a final primer concentration of 75 nM. The reaction was conducted with an initial denaturation step at 95°C for a duration of 5 minutes, followed by 39 cycles of denaturation at 95°C for 10 seconds, annealing at 60°C for 30 seconds, and extension at 72°C for 30 seconds. The PCR products were analyzed by melt-curve analysis, ranging from 65 to 95°C with an increment of 0.5°C for a duration of 5 seconds at each step. The amplification efficiencies were calculated with the CFX manager software. Afterwards, the amplification products were analyzed by gel electrophoresis. Only primer pairs that showed the expected amplification product size in the gel electrophoresis, one specific melt-curve peak, and sufficient amplification efficiency (85%–115%) were selected for the following analysis.

### Gene expression analysis

The expression of the 90 CGs was analyzed using the BioMark HD high-throughput qPCR system (Standard BioTools, USA). The genes *ACT7*, *EF1a*, *EF1b*, *TUBB*, and *UBE210* (Table S2) were used as reference genes according to Weiß *et al.* [23]. Equal amounts of 57 cDNA samples isolated from ‘M.26’ roots grown in ARD affected soils were mixed and diluted 1:10, 1:100, and 1:1000 in series to be used as standards for the calculation of amplification efficiencies. A total of 338 cDNA samples with two technical replicates each were analyzed with the 96.96 Dynamic Array™ IFC (Standard BioTools, USA) following the manufacturer’s instructions. First, 1.25 μl of the cDNA were pre-amplified with the 5× PreAmp Master Mix (Standard BioTools, USA) and 500 nM pooled primers filled up to 5 μl with nuclease-free water. The PCR was performed with an initial denaturation at 95°C for 10 minutes, followed by 14 cycles of 95°C for 15 seconds and 60°C for 4 minutes. Following this, the pre-amplified cDNAs were purified with exonuclease I (New England Biolabs GmbH, Germany), diluted 1:5 with DNA suspension buffer pH 8.0 (Alpha Teknova, USA) and stored at −20°C until use. For the sample-mix, 2.7 μl of the pre-amplified and diluted cDNA was mixed with 3 μl 2× SsoFast EvaGreen supermix with low ROX.

(Bio-Rad Laboratories, USA) and 0.3 μl 20× DNA binding dye sample loading reagent (Standard BioTools, USA). The assay-mix consisted of 0.5 mM primer mix, 2.5 μl 2× assay loading reagent (Standard BioTools, USA), and 2.25 μl DNA suspension buffer pH 8.0 (Alpha Teknova, USA). The qPCR was performed with 5-μl sample-mix and assay-mix per inlet in 96.96 Dynamic Array™ IFCs.

(Standard BioTools, USA). The cycling program was as follows: 95°C for 1 minute, 30 cycles of 96°C for 5 seconds and 60°C for 20 seconds plus melting curve analysis from 60°C to 95°C with an increment of 1°C for a duration of 3 s at each step.

### Data analysis

The normalization of the growth data of each biotest set was done by multiplying it by a normalization factor derived from the growth data of the plants grown in peat substrate, using the following formula:


$$ \mathrm{Normalization}\ \mathrm{factor}\ \left(\mathrm{Biotest}\ \mathrm{x}\right)=\frac{\mathrm{Mean}\ \left(\mathrm{all}\ \mathrm{Biotests}\right)}{\mathrm{Mean}\ \left(\mathrm{Biotest}\ \mathrm{x}\right)} $$

The normalized growth reduction for each growth parameter (SDM, SFM, and RFM) was calculated as a percentage of the reduction in the ARD variant of a soil compared to the control variant of the same soil.

The initial gene expression data analysis was performed with the Fluidigm Real-Time PCR Analysis program version 4.8.1 (Standard BioTools, USA). To ensure the quality of the measurements, each amplification curve was scored based on how well it compared to an ideal curve and all samples that scored below the recommended threshold set by the Fluidigm program were excluded from further analysis. Samples showing unspecific amplification in the melt curve analysis were excluded as well. Additionally, the Fluidigm program calculated the amplification efficiencies of each primer pair. The stability of the reference genes was analyzed with RefFinder [38] and based on the results, the gene *UBE210* was excluded.

Next, the Fluidigm program calculated the ΔCt, ΔΔCt and fold change values for every sample according to the following formulas:


$$ \Delta \mathrm{Ct}=\mathrm{Ct}\ \left(\mathrm{candidate}\ \mathrm{gene}\right)-\mathrm{average}\ \mathrm{Ct}\ \left(\mathrm{reference}\ \mathrm{gene}\mathrm{s}\right) $$

$$ \Delta \Delta \mathrm{Ct}=\Delta \mathrm{Ct}\ \left(\mathrm{ARD}\ \mathrm{sample}\right)-\Delta \mathrm{Ct}\! \left(\mathrm{corresponding}\ \mathrm{control}\ \mathrm{sample}\right) $$

$$ \mathrm{Fold}\ \mathrm{change}={2}^{-\Delta \Delta \mathrm{Ct}} $$

Afterwards, statistical analysis was performed using R version 4.4.1 [55] and the log_2_FC was calculated for each sample. The correlation between the log_2_FC and the plant growth data (SDM_red, SFM_red, RFM_red) for each gene was analyzed by Spearman correlation.

Additionally, the calibrated normalized relative quantification of gene expression was determined with the qBase+ software version 3.4 (CellCarta, Canada). A paired Wilcoxon signed rank test was used to calculate the significance of the difference in gene expression between the ARD and control samples.

## Supplementary Material

Web_Material_uhag140

## Data Availability

The datasets presented in this study can be found in the supplementary material or are available in the BonaRes Repository [1, 2, 35].
