# Correction to: Establishing a biomarker set for the diagnosis of apple replant disease using qPCR

**DOI:** 10.1093/hr/uhag391

**Published:** 2026-10-05

This is a correction to: Luisa Baader, Susan Schröpfer, Stefanie Reim, Traud Winkelmann, Nils Orth, Jiem Krueger, Georg Guggenberger, Jens Boy, Jacqueline Kaldun, Eva Lehndorff, Nele Meyer, Kristin Hauschild, Henryk Flachowsky, Establishing a biomarker set for the diagnosis of apple replant disease using qPCR, *Horticulture Research*, Volume 13, Issue 8, August 2026, uhag140, https://doi.org/10.1093/hr/uhag140

The following changes have been made to the originally published paper.

The list has been increased and changed in order by the addition of two co-authors: Dr. Benye Liu and Dr. Ludger Beerhues. The affiliations list has been emended to include: “Technische Universität Braunschweig, Institute of Pharmaceutical Biology, Braunschweig, Germany”. All enumerations in the author and affiliations lists have been emended and respectively incremented where needful due to these additions.

The statement: “The authors thank Dr. Benye Liu (Technical University Braunschweig, Institute of Pharmaceutical Biology, Braunschweig, Germany) for providing the sequences of the putative phytoalexin genes.” has been deleted from **Acknowledgments; Author contributions** section has been duly expanded and initials “L.B” emended to “L.Ba” and L.Be” where needful.

