# Supplementary material for: Establishing a biomarker set for the diagnosis of apple replant disease using qPCR
Source: Hortic Res. 2026 Apr 16;13(8):uhag140. doi: 10.1093/hr/uhag140 (PMC13452168; doi:10.1093/hr/uhag140)
Supplement: Supplementary file 1 [file web_material_uhag140.zip › Figure S2 Relative expression values of three example genes for each soil sample.pdf]

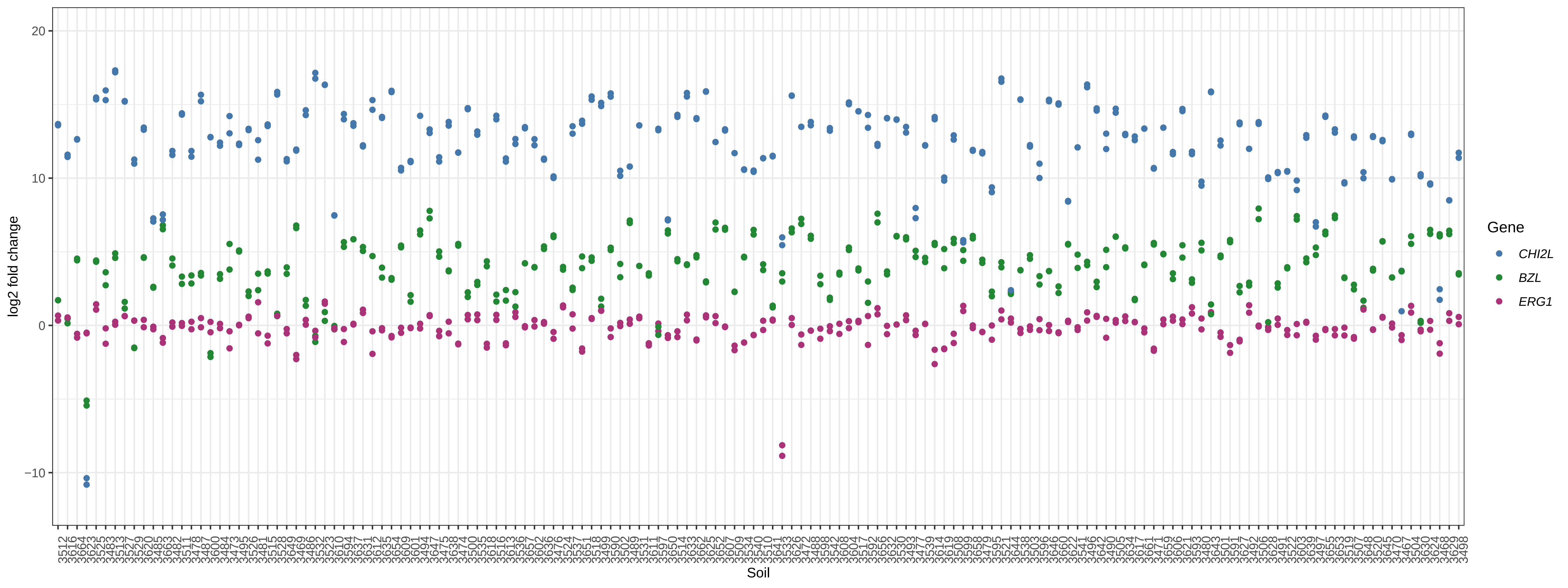

20
10
0
−10
log2 fold change
Soil
Gene
CHI2L
BZL
ERG1
