# Supplementary material for: Establishing a biomarker set for the diagnosis of apple replant disease using qPCR
Source: Hortic Res. 2026 Apr 16;13(8):uhag140. doi: 10.1093/hr/uhag140 (PMC13452168; doi:10.1093/hr/uhag140)
Supplement: Supplementary file 1 [file web_material_uhag140.zip › Figure S3 Consistency of upregulation across the different soils of two example genes.pdf]

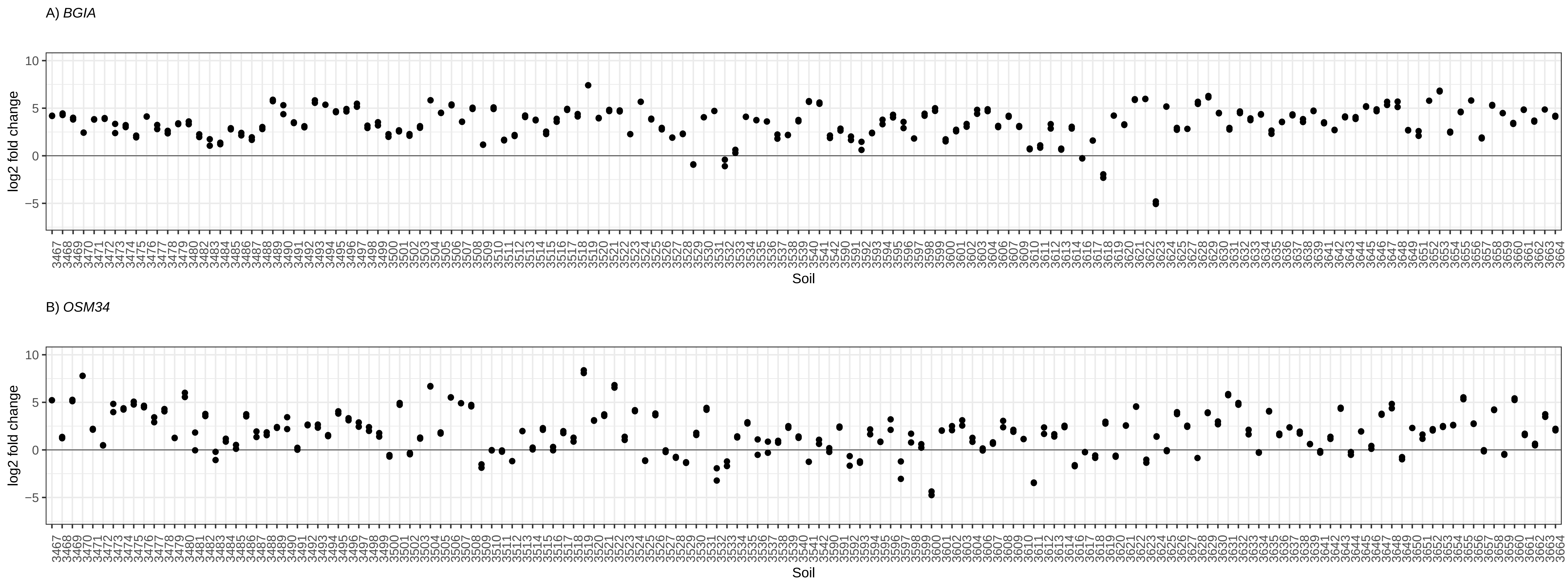

A) *BGIA*
10
5
0
−5
log2 fold change
Soil
B) *OSM34*
10
5
0
−5
log2 fold change
Soil
